# A Comprehensive Review on the Importance of MiRNA-206 in the Animal Model and Human Diseases

**DOI:** 10.2174/1570159X21666230407124146

**Published:** 2023-05-03

**Authors:** Wang Qi, Wei Guan

**Affiliations:** 1 Department of Pharmacology, The First People's Hospital of Yancheng, Yancheng, 224000, Jiangsu, China;; 2 Department of Pharmacology, Pharmacy College, Nantong University, Nantong, 226001, Jiangsu, China;; 3 School of Medicine, Nantong University, Nantong, China

**Keywords:** MiRNA, miR-206, biomarkers, human diseases, expression levels, Alzheimer’s disease

## Abstract

MicroRNA-206 (miR-206) is a microRNA that is involved in many human diseases, such as myasthenia gravis, osteoarthritis, depression, cancers, *etc*. Both inhibition effects and progression roles of miR-206 have been reported for the past few years. High expression of miR-206 was observed in patients with osteoarthritis, gastric cancer and epithelial ovarian cancer compared to normal people. The study also showed that miR-206 promotes cancer progression in breast cancer patients and avascular necrosis of the femoral head. Meanwhile, several studies have shown that expression levels of miR-206 were down-regulated in laryngeal carcinoma cell multiplication, as well as in hepatocellular carcinoma, non-small lung cancer and infantile hemangioma. Moreover, miR-206 was up-regulated in the mild stage of amyotrophic lateral sclerosis patients and then down-regulated in the moderate and severe stages, indicating that miR-206 has the double effects of starting and aggravating the disease. In neuropsychiatric disorders, such as depression, miR-206 also plays an important role in the progression of the disease; the level of miR-206 is most highly expressed in the brains of patients with depression. In the current review, we summarize the role of miR-206 in various diseases, and miR-206 may be developed as a new biomarker for diagnosing diseases in the near future.

## 1 INTRODUCTION

MicroRNAs (miRNAs) represent a type of endogenous, evolutionarily conserved, non-coding small RNAs, which are made up of about 20-22 nucleotides in size [1]. The first two known miRNAs were discovered over 35 years ago in the nematode Caenorhabditis elegans with the identification of the developmental regulator lin-4 and human miRNA let-7. From then on, plenty of miRNAs have been studied by scientists in various fields, like plants, mammals and some viruses [2]. MiRNAs are derived from primary miRNA transcripts (pri-miRNAs) and high efficiency and precision excisions from imperfect stem-loop residing in the pri-miRNAs [3], which are different from other kinds of small RNAs, such as PIWI-interacting RNA (piRNA), small interfering RNA (siRNA), and transfer RNA-derived small RNA (tsRNA) [4-6]. The great majority of miRNAs are transcribed by the DNA-dependent RNA polymerase II and processed into pre-miRNA composed of 70 to 100 ribonucleotides with the help of a ribonuclease III (Drosha) [7]. Finally, Dicer, another ribonuclease III, recognizes and splices it into a mature 20-24 nucleotides (nt) miRNA [8].

An accumulating body of research indicates that miRNAs play important roles in gene regulation and phenotype development by targeting messenger RNA (mRNA) transcripts. According to studies, miRNAs can potentially regulate more than 60% of human protein-coding genes [9]. Nowadays, more than 2,000 miRNAs have been illustrated to have a stronger relationship with about one-third of the genes in the human genome [10]. Moreover, miRNAs often induce gene overexpression or silencing by binding to the complementary sequences in the 3’-untranslated regions (UTRs) of targeted mRNAs, and therefore miRNAs may serve as the post-transcriptional gene regulators to strengthen or weaken the gene expression and control cellular function [11, 12]. MiRNAs can be packaged and released in extracellular vehicles (EVs), serving as a messenger for communication and regarded as diagnostic and predictive biomarkers and therapeutic targets for diseases.

MiR-206, known as a muscle-specific miRNA, plays a vital role in the process of myogenesis [13]. The first discovery of miR-206 was identified as a special expression in the skeletal muscle, so the original studies on miR-206 were limited to the physiological and pathological processes of skeletal muscles. The development of miR-206 in skeletal muscles and its roles in related diseases are explained in detail. MiR-206 is the focal point of this review. Although researchers have done much clinical and basic science research on miRNA, the pathological mechanisms of miRNA that correlate with disease progression remain unclear. In recent years, it has been found that the high expression of miR-206 is not only in skeletal muscles but also expressed highly in other organs, such as lungs, hearts and cerebrums, as well as several forms of tumor tissues (breast cancer, ovarian cancer, and gastric cancer). More importantly, miR-206 regulates some physiological and pathological processes of tissues, highlighting a need to research further on miR-206. Therefore, in this review, the research progress of miR-206 has been discussed.

In the current review, we first introduce the structure and biological characteristics of miR-206; then, the relationship between miR-206 and tumor tissues and the roles of miR-206 in organs are expounded in more detail.

## 2 METHODS

We searched databases, such as PubMed (https://pubmed.ncbi.nlm.nih.gov), Springer (https://link.springer.com), ScienceDirect (https://www.sciencedirect.com), and Scopus (https://www.scopus.com/home.uri), by November 1^st^, 2022, with no time or language restriction. The searches comprised keywords, such as ‘miRNA’, ‘miR-206’; ‘disease’, ‘biomarkers’, ‘animal model’, ‘mechanisms’, ‘cancer’, ‘human disorders’, ‘pathway’, ‘target genes’, ‘malignant’ and ‘non-malignant’. We retrieved up to 952 articles based on a rigorous selection criterion, and only 155 articles were extensively reviewed. We selected articles only addressing the role of miR-206 in disease and the molecular mechanism in animal models, with the employed approach being miRNA analysis and/or profiling. Inclusion criteria were studies on the expression of miR-206 in animal models and common diseases. Exclusion criteria were studies on miRNAs other than miR-206 were excluded, and unfrequent diseases were also ruled out.

## 3 THE STRUCTURE AND BIOLOGICAL CHARACTERISTICS OF miR-206

**Fig. (1):**
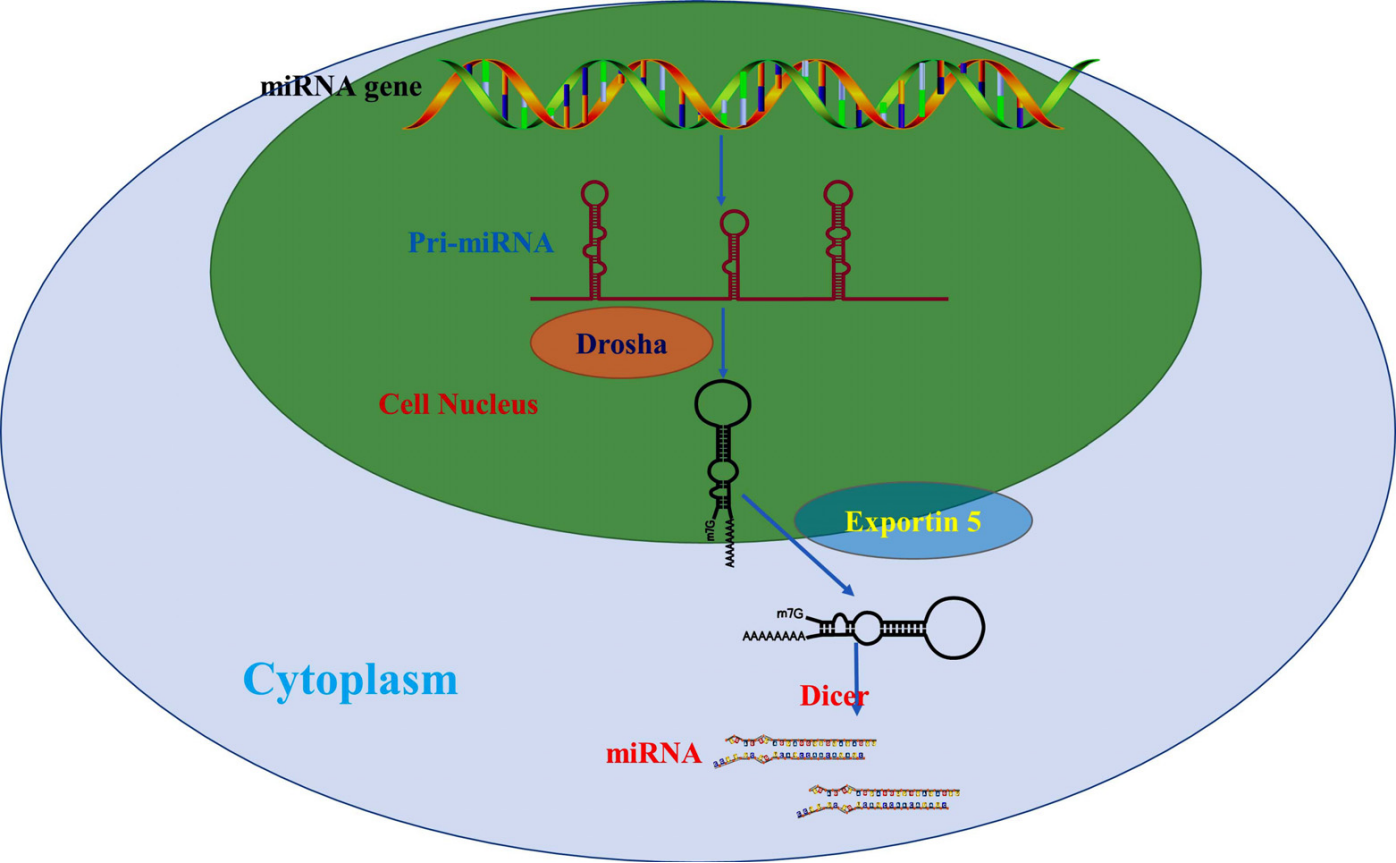
The canonical biogenesis of miR-206 is a complex pathway, which can be divided into two steps: nuclear and cytoplasmic steps. MiRNAs are derived from pri-miRNAs and processed into pre-miRNA with the help of Drosha. Finally, Dicer recognizes and splices it into a mature miRNA.

As a member of the miR-1 family, miR-206 is located between IL-17 and PKHD1 genes in humans [14]. The canonical biogenesis of miR-206 is a complex pathway, which can be divided into two steps: nuclear and cytoplasmic steps (Fig. **1**). First, miR-206 is transcribed by the RNA polymerase II (Pol II) and produces a hairpin intermediate called “pri-miR-206” [15, 16]. Pri-miR-206 is then recognized by the molecule of Drosha and DiGeorge syndrome critical region gene 8 (DGCR8). Drosha is a kind of RNase III enzyme with two RNase III domains, and DGCR8 is the accessory factor of Drosha [17]. In a second step, it forms a stem-loop named “pre-miR-206” with the Drosha by cleaving two strands of the stem in the pri-miR-206 hairpin [18]. Subsequently, pre-miR-206 (in the nucleus) is transported to the cytoplasm with the assistance of Exportin 5 and Ran guanosine triphosphate (RanGTP) [19]. Interestingly, pre-miR-206 is further identified by an endonuclease with two RNase III domains named Dicer in the cytoplasm; Dicer can also cut off the terminal loop from the hairpin-like Drosha and be further processed into mature double-stranded RNA molecules (miRNA-miRNA* duplex) [20, 21]. Finally, the mature form of miR-206 is retained, and incorporate miR-206 into the protein complex RNA-induced silencing complex (RISC). When miR-206 is included in the RISC, the mature miR-206 recognizes target mRNAs by base-pairing interactions between nucleotides 2 and 8 of the miRNA (the seed region) and complementary nucleotides in the 3′-untranslated region (3′-UTR) of mRNAs, leading to translational repression or mRNA decay, thus playing a major role in biological characteristics.

MiR-206 is located on the human chromosome 6p12.2, the mice chromosome 1p, and the rat’s chromosome 9p. The sequence of miR-206 is highly conserved in different species [22, 23]. Lagos-Quintana first discovered human sequences of miR-206 in 2003 according to homology analysis of confirmed sequences in mice [24]. Landgraf subsequently confirmed the sequences of miR-206 in humans in 2007 [25]. The studies above indicated that miR-206 might play an important role in the primary physiological functions and pathological processes of mammals.

MiR-206 belongs to one of the members of the “muscle-specific miRNA (myomiR)” family; its other members include miR-1 and miR-133 [26]. There are significant similarities between their sequence, indicating that they have an identical or similar target gene. MiR-206, miR-1 and miR-133 construct three gene clusters with the opposite effects [27]. MiR-206 may bind with more than a hundred target genes, such as BDNF (brain-derived neurotrophic factor, BDNF) [28], G6PD (Glucose-6-phosphate dehydrogenase, G6PD) [29], GATA4 (GATA-binding protein 4, GATA4) [30], HDAC4 (histone deacetylase 4, HDAC4) [31], *etc*., through the database retrieval of TargetScan and miRDB. We also found various binding action sites of a target gene and miR-206. It was also observed that the more binding sites involved, the more degree was suppressed by miR-206. Thus, it revealed miR-206 was in a key position to regulate the networks of complex biological macromolecules. MiR-206 appears to play an important role in the physiology of polygene regulation and can be used for clinical purposes, such as diagnosis and prognosis of tumors in the future. It seems to be one of the most attractive miRNAs.

## 4 THE BIOLOGICAL FUNCTION OF miR-206

Several studies have assessed the expression of miR-206 in different types of disease and found the molecular mechanism of involvement of this miRNA in particular ailments.

Table **1** summarizes the role of miR-206 in non-malignant and malignant conditions.

### 4.1 The Biological Function of miR-206 in Non-malignant Disease

#### 4.1.1 MiR-206 in Skeletal Muscle Development

Skeletal muscle is an important component of the human body and plays a crucial role in the control of presystemic metabolism in mammals. Research has shown that four myomiRs, miR-1, miR-206, miR-133a and miR-133b, take part in local skeletal muscle communication [32]. Moreover, they are involved in the process of myogenesis by relying on their expression activity of transcription factors [33]. It was found that miR-133a and miR-133b promote myoblast proliferation and muscle growth, whereas miR-1 and miR-206 promote myoblast differentiation and regeneration *in vitro* and *in vivo* [14, 34].

MiR-206, one of the skeletal muscle-specific microRNAs, plays a strong part in myogenesis. In the rat models of skeletal muscle injury, the levels of serum miR-206 significantly increased after treatment with TMPD (2,3,5,6-tetramethyl-p-phenylenediamine, TMPD) as compared to control rats. MiR-206 may serve as a highly specific biomarker for preclinical analysis in the drug-induced skeletal muscle injuries of rats [35]. It is interesting that the miR-206 family is not essential for myogenesis and is instead a modulator of optimal differentiation of skeletal myoblasts [36].

Recent research has shown that exosomes carrying muscle-specific miRNAs (miR-206) are elevated in the blood of muscle disease patients. It reveals a novel contribution of the molecules in the communication of tissues by the discovery of miR-206 within exosomes and confirms its functional role in the recipient target cells [37-39].

**Fig. (2):**
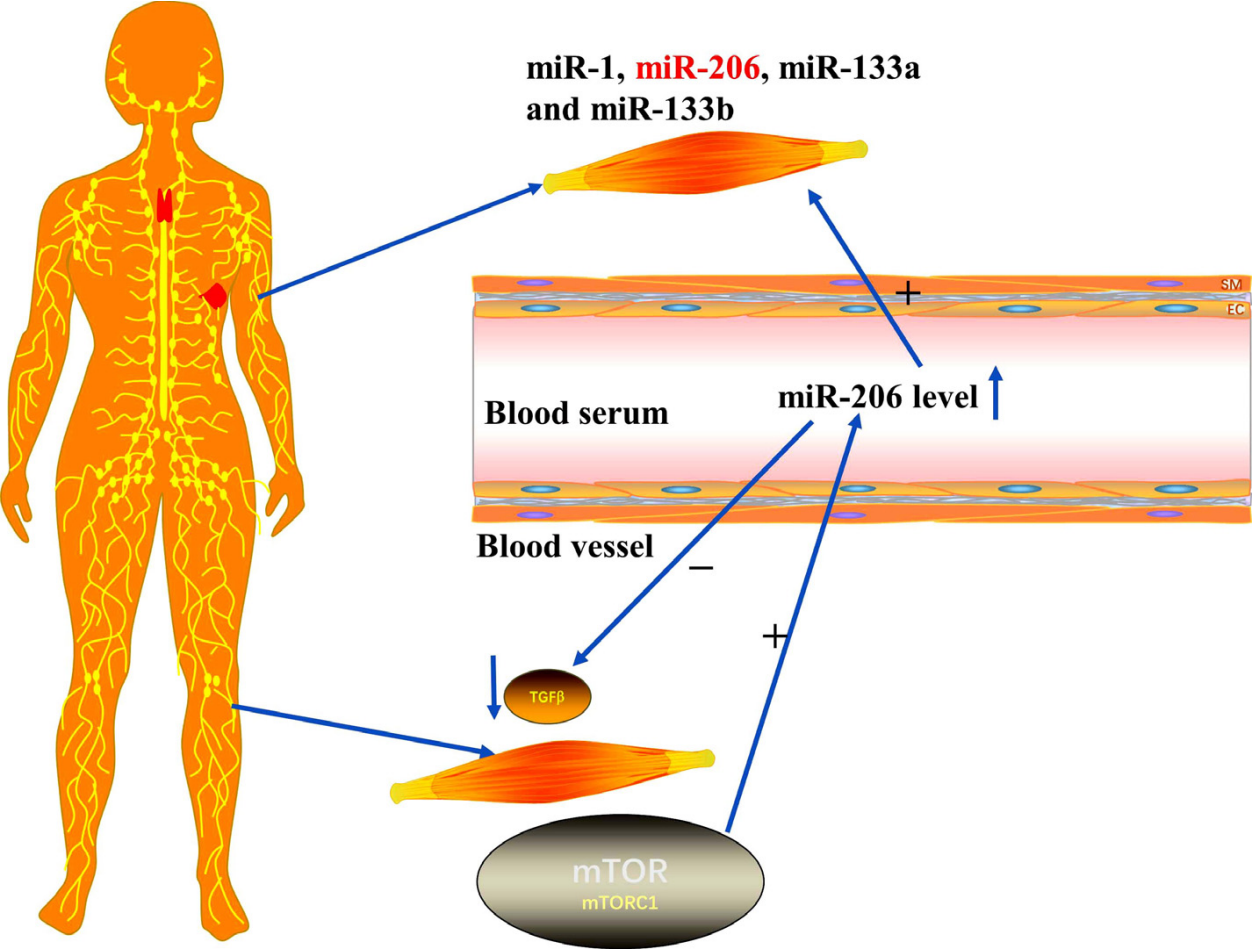
MiR-1, miR-206, miR-133a, and miR-133b take part in local skeletal muscle communication; the levels of serum miR-206 significantly increased after treatment with the therapeutic drug in skeletal muscle injury, and the action of miR-206 was linked by the upstream and downstream relationships.

An interesting clue is that miR-206 appears to be directly or indirectly regulated by the mammalian target of rapamycin (mTOR) [33], the important regulator of muscle maintenance and skeletal myogenesis and the main mediator of cellular nutrient sensing [40]. Zhang *et al*. reported that the kinase-dependent mTOR pathway affects the expression of the miR-206 through regulation of the myogenic transcription factor MyoD in a model of nutrient-mTOR-myomiR signaling in skeletal myogenesis [41]. In this model, mTOR is inactive and unable to induce MyoD synthesis under the low nutrient conditions of glucose starvation and amino acids, which can, in turn, lower the expression of miR-206. It is widely known that increased inflammation and oxidative stress have been closely linked with sarcopenia and malnutrition [42-44]. Significantly, the downregulation of miR-206 was observed in the muscle of patients with inflammatory myopathy [45]. Literature data report that miR-206 participated in the skeletal muscle *via* regulating the expression of myostatin induced by inflammation and oxidative stress. Myostatin is a member of the transforming growth factor beta (TGF-β) superfamily, which inhibits skeletal muscle growth [46]. More importantly, studies suggest that miR-206 is not associated with sarcopenia. This is probably because miR-206 expression is regulated by different upstream signals, and also, miR-206 regulates the myogenic program by activating different downstream targets. The action of miR-206 is linked by the upstream and downstream relationships (Fig. **2**).

#### 4.1.2 MiR-206 in Alzheimer’s Disease

**Fig. (3):**
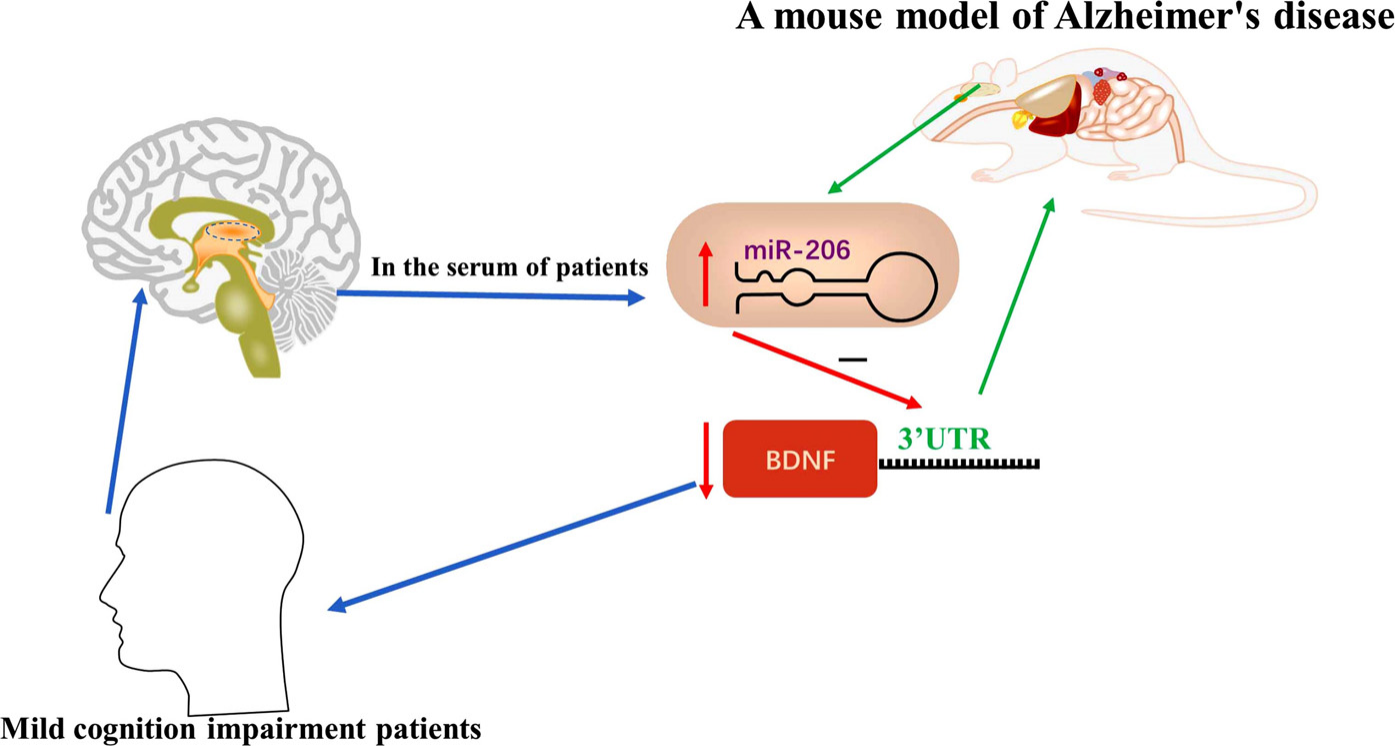
The level of serum miR-206 was elevated in patients with mild cognition impairment (MCI) and promoted the AD symptom *via* inhibiting the level of BDNF in AD model brain tissue of mice.

As a family of short non-coding RNAs, miRNA-related pathways participate in various diseases, including neurodegenerative diseases. MiR-206 plays an important role in the regulation of AD progression. Alzheimer’s disease (AD) is a neurodegenerative disorder related to age and one of the leading causes of disability and mortality in late life. Unfortunately, there is currently no known effective cure for the disease [47]. It is estimated that the morbidity related to AD will rapidly increase to 75.6 million by 2030 throughout the world and 135.5 million by 2050 [48]. Compared with normal people of the same age, the level of serum miR-206 was elevated in mild cognition impairment (MCI) patients (Fig. **3**). The miR-206 levels were also up-regulated in the hippocampal tissue and plasma of embryonic APP/PS1 transgenic mice, and Tg2576 mice brain as well as the temporal cortex of human AD brains [49, 50]. Lee and colleagues showed that miR-206 promoted the detrimental effect of Aβ42 on the brain-derived neurotrophic factor (BDNF) *via* inhibiting the level of BDNF [49, 50]. Donepezil has been approved for treating AD in the clinic, and miR-206 is a target of donepezil, so miR-206 inhibitor is able to relieve the detrimental effects of Aβ42 [51].

Cerebrospinal fluid (CSF) biomarkers for early and differential Alzheimer’s disease diagnosis have been the hot spots in recent years. CSF is in the continuum of the brain. Cerebrospinal fluid can reflect central neuropathological features of brain diseases in AD patients [52], so it is an attractive source of biomarkers of AD in clinical practice. Recently, emerging studies have suggested that CSF contains a large number of miRNAs, such as miR-27a-3p, miR-125b, miR-206, miR-15a-5p, and miR-29a [50, 53-56]. The expression levels of the above miRNAs were found to be increased in the CSF of AD patients, but interestingly, the levels of some miRNAs, such as miR-214-3p, miR-210 and miR-384 were decreased as compared to normal people [57-59]. Among them, the up-regulated expression of miR-206 is the main cause of AD pathology *via* suppression of the neuroprotective factor BDNF. The results were consistent with the above research [50].

New research has examined the relationship between miR-206 and BDNF. Shao and Xu presented a different view that the level of miR-206-3p (a subtype of miR-206) was decreased in AD modelling of mice (brain stereotactic injection of Aβ25-35) as compared to normal mice, while the expression level of miR-206-3p (miR-206-3p mimics treatment) increased significantly compared to AD model group [60]. Thus, miR-206-3p exerted a neuroprotective effect on neuronal morphology and improved the cognitive ability and memory of AD mice by upregulating BDNF.

In brief, miR-206 is a critical biomarker with high predictive accuracy in the pathogenesis process of AD.

#### 4.1.3 MiR-206 in Mice Models of Depression

Depression is one of the most common neuroscience diseases associated with cognitive impairment and a major cause of death and disability [61]. It has been found that over 120 million patients do not adequately respond to antidepressant treatments, and casually increasing the dose of medicine often leads to significant side effects [62]. Thus, it is necessary to research new therapeutic biomarkers of depression in clinical treatment since they may be used as useful targets for the development of new drugs.

It has been well elucidated that miRNAs have a precise role in the expression of coding genes, cell proliferation and the central nervous system in literature [63, 64]. Furthermore, several miRNAs, such as miR-206-3p and miR-124, have been reported to be associated with the pathogenesis of depression [28, 65]. For example, Guan and his research group confirmed the link between miR-206-3p and depression; the level of miR-206-3p, not miR-206-5p, was markedly elevated in the hippocampus of CSDS-induced mice. Genetic overexpression of miR-206-3p aggravated the depression-like behaviors and neurogenesis damage of normal mice, while genetic knockdown of miR-206-3p relieved the neurological damage and produced antidepressant effects in behavioral tests in the depression model of mice [28].

MiR-206 also participates in the maladaptive impulsive aggression of post-weaning social isolation mice [66]. Maladaptive impulsive aggression is a social problem, which brings a heavy burden to society and families. Research suggests that the level of miR-206 was higher in socially isolated (SI) mice as compared to group housing (GH) mice, and stereotactic injection of an antagomir of miR-206 (AM206) increased BDNF expression and decreased stress-induced attack behavior in SI mice. Furthermore, overexpression of miR-206 induced attack behavior in GH mice. BDNF plays a crucial role in neuroprotection [67] and neuroplasticity [68], which is also associated with brain development. It was found that BDNF expression is required to inhibit miR-206, thus reducing aggression behaviour.

**Fig. (4):**
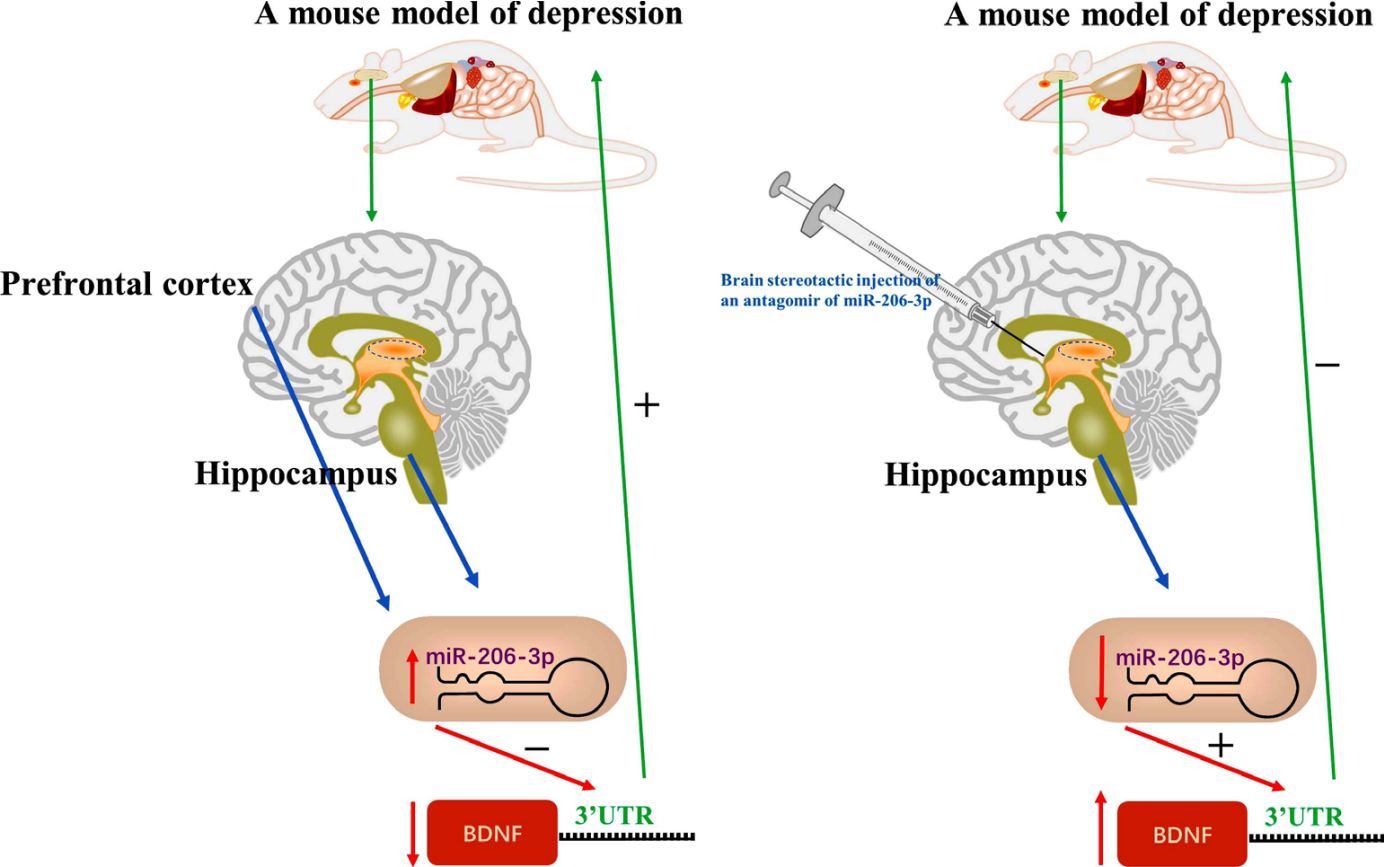
Enhanced miR-206-3p levels and decreased BDNF expression were observed in both the hippocampus and mPFC of PS mice, and brain stereotactic injection of an antagomir of miR-206 increased BDNF expression and decreased stress-induced attack behavior in SI mice.

The incidence of depression is higher in women (especially in pregnant women) than in men [69], and depression seems to have a link with gender. Some studies have indicated that pregnant women in a hostile environment are susceptible to depression [70]. Miao research team pointed out that enhanced miR-206-3p levels and decreased BDNF expression were observed in both the hippocampus and medial prefrontal cortex (mPFC) of pregnant stressed (PS) mice [71]. On the contrary, in the amygdala of the same PS mice, BDNF expression was elevated while the miR-206-3p level was decreased. These results suggest that the changes between miR-206-3p and BDNF expression in the hippocampus, mPFC, and amygdala of PS mice induce the onset of depression (Fig. **4**).

### 4.2 The Role of miR-206 in Malignant Disease

#### 4.2.1 MiR-206 in Hepatic Disease

According to the World Health Organization (WHO) report, hepatocellular carcinoma (HCC) is the major and most common disease and one of the deadliest forms of cancer all over the world [72, 73]. HCC is a disease with a disorder of the cell cycle and uncontrolled growth due to hereditary factors and environmental factors, such as typically chronic hepatitis B and C. Alcoholic liver disease is a key element among the factors. New evidence suggests that the abnormality of gene affects the initiation and progression of HCC [74].

**Fig. (5):**
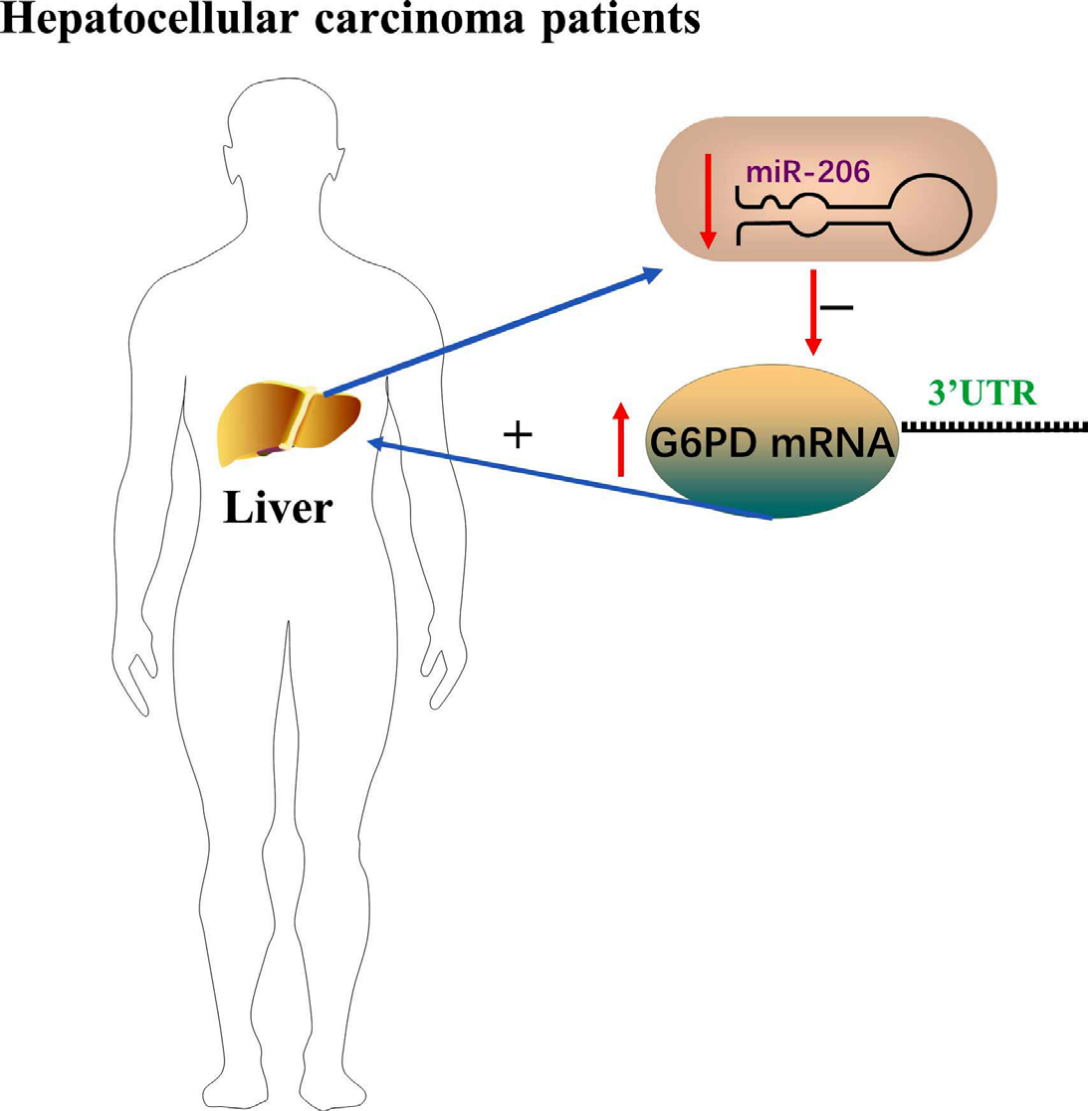
MiR-206 was dysregulated in HCC tissues of HCC patients and directly targeted G6PD and upregulated the expression of G6PD to promote hepatocellular carcinoma cell growth.

Recently, researchers have shown that miR-206 is involved in the carcinogenesis and progression of various cancers and acts as a tumor suppressor [75, 76]. Yang and his research group and Wang and his research group demonstrated that miR-206 could suppress cancer cell proliferation and promote apoptosis by targeting the genes involved in oncogenic signal pathways [75, 76]. MiR-206 is dysregulated in HCC tissues of HCC patients, in turn, causes the low expression level of miR-206 in HCC. In the end, miR-206 directly targets glucose-6-phosphate dehydrogenase (G6PD) and downregulates the expression of G6PD to inhibit hepatocellular carcinoma cell growth (Fig. **5**) [29].

However, some researchers hold different views on the role of miR-206 in HCC patients. Wu and his colleagues indicated that the levels of miR-206 were significantly higher in plasma samples of hepatocellular carcinoma patients compared to healthy subjects through high-throughput small RNA sequence and quantitative polymerase chain reaction (qPCR) analysis [77]. MiR-206 can be used as an effective biomarker to screen patients at risk of HCC. MiR-206 may play a dual role in the plasma and tissues of HCC patients, thus requiring further studies.

#### 4.2.2 MiR-206 in Heart Failure

Heart disease is the leading cause of death in the United States, with approximately 6 million adults suffering from heart failure (HF) [78]. HF is a difficult-to-cure heart disease with substantial health and economic impacts, causing serious consequences [79]. Although effective drugs for the treatment of HF have been used in the clinic, the mechanisms involved in HF are not clearly understood, and there is still a lack of clinically available drugs to completely heal HF patients so far.

**Fig. (6):**
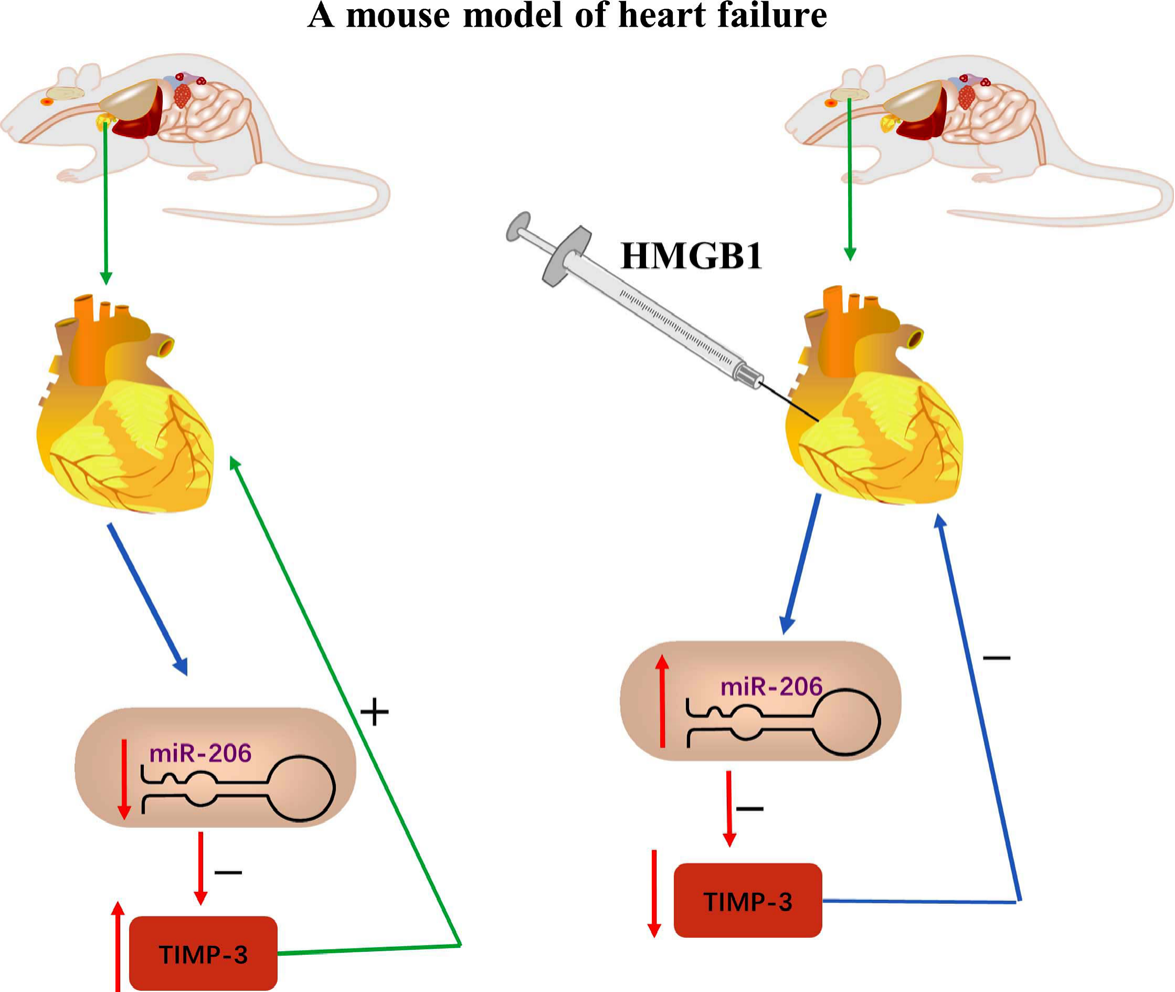
HMGB1 intramyocardial injection improved LV function and remodelling, and these effects were associated with miR-206 overexpression and miR-206-mediated inhibition of TIMP-3.

The latest evidence suggests that miRNAs participate in a large and complex regulatory network of gene expression of the majority of the protein-coding genes [80]. Currently, research findings show that miRNAs play a crucial role in the pathogenesis of heart failure [81]. Some miRNAs, especially miR-206 expression, were found to be higher in control hearts than in the heart of infarcted mice [82]. Limana and colleagues indicated that high mobility group box-1 protein (HMGB1) injected into peri-infarcted regions of chronically failing hearts in mice attenuated left ventricular (LV) remodelling and, at the same time, enhanced LV function [82]. The effects of HMGB1 on enhanced efficacy of LV function were associated with miR-206 overexpression and miR-206-mediated suppression of tissue inhibitor of metalloproteinase 3 (TIMP-3). Interestingly, the study also noted that the level of miR-206 was increased in the failing LV of mice compared to normal mice; HMGB1 did not modulate miR-206 and only further increased miR-206 expression *in vitro* (Fig. **6**).

MiR-206 may play a double role in the endogenous or exogenous system. The lower expression of endogenous miR-206 attenuated yes-associated protein (YAP)-induced cardiac hypertrophy and survival in cardiomyocytes of mice, suggesting that miR-206 plays a critical role in mediating YAP function [83]. YAP is known as a transcription co-factor and promotes cardiac regeneration and remodeling after myocardial infarction in mice [84]. Research by Yang *et al*. contributed to new information that in Tg-206-SPONGE mice, downregulation of endogenous miR-206 aggravated myocardial ischemia/reperfusion (I/R) injury compared to NTg mice, whereas restoration of miR-206 levels with exogenous miR-206 counteracted the progression to heart failure and protected against I/R injury [83].

However, some researchers have a different opinion on the role of miR-206 in the acute myocardial infarction (AMI) model of rats. Studies confirmed that down-regulation of miR-206 increased cardiomyocytes apoptosis *in vitro*; the expression of miR-206 was decreased in the infarcted myocardial areas of rats compared to non-infarcted areas, while overexpression of miR-206 decreased cardiomyocytes apoptosis. The results suggested the protective effect of miR-206 against cardiomyocytes apoptosis on the AMI model of rats *in vitro* [85].

#### 4.2.3 MiR-206 in Lung Cancer

Lung cancer is one of the principal diseases threatening human health worldwide. It is estimated that approximately 1.3 million people show symptoms of illness every year. Non-small lung cancer (NSCLC) is the major type of lung cancer with a higher 5-year mortality rate (85%), indicating that the vast majority of lung cancer-related mortality is caused by NSCLC [86]. The biggest cause of NSCLC therapy failure is metastasis, although some progress has been made in clinical therapeutics of NSCLC, such as radiotherapy, surgical therapy, chemotherapy and drug targeting therapy [87].

**Fig. (7):**
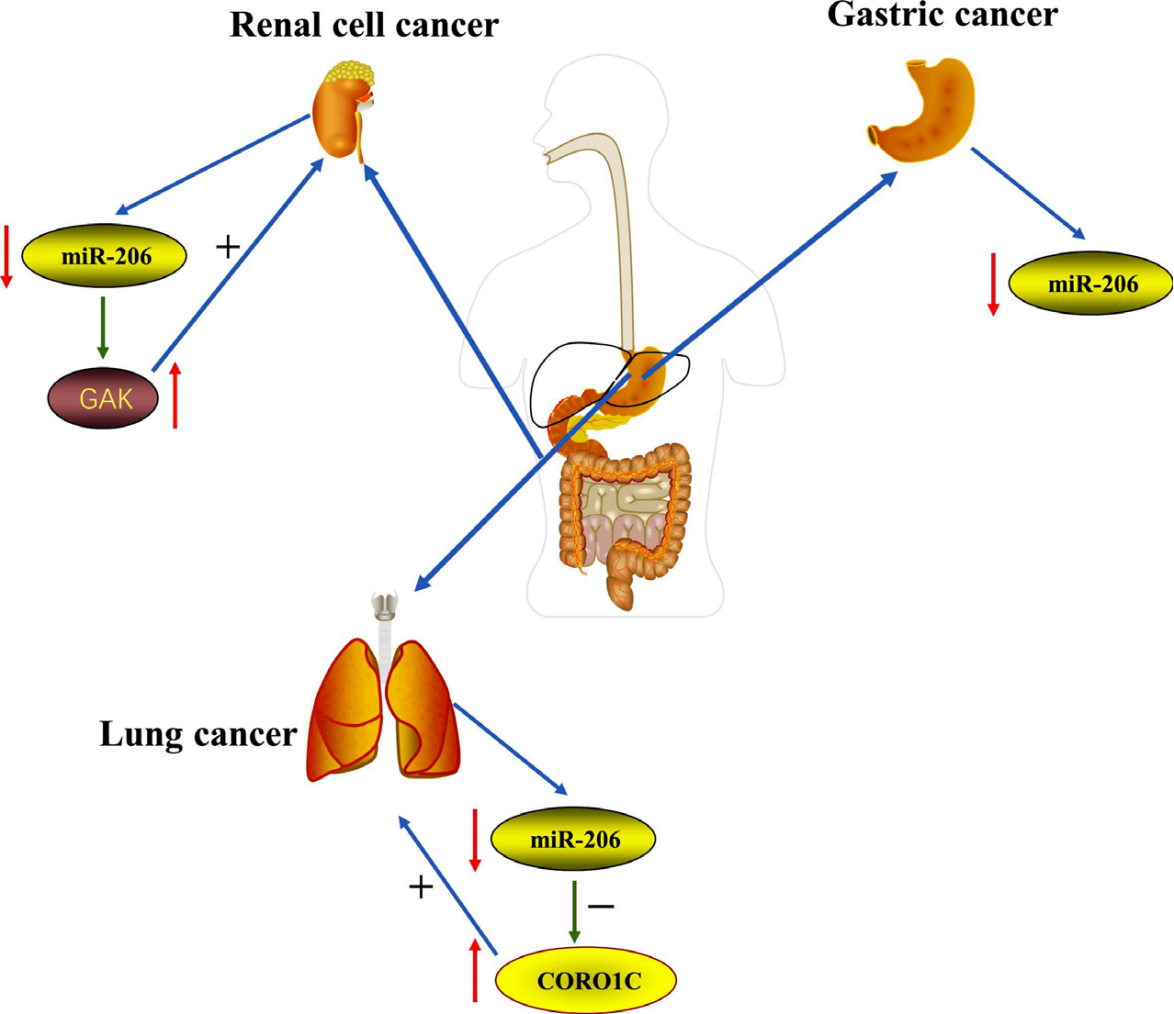
The expression level of miR-206 was decreased in lung cancer cells and negatively regulated CORO1C and then restrained the proliferation, migration and invasion of A549 cells. The level of miR-206 was noticeably decreased in advanced patients with gastric cancer compared to normal people. MiR-206 inhibited RCC cell growth partly by targeting GAK in RCC patients.

Accumulating evidence has proven that many miRNAs, including miR-204, miR-34a and miR-206, play a key role in NSCLC metastasis [88-90]. Besides NSCLC, miR-206 has an inhibitory effect on many cancers, such as gastric cancers [91], colorectal cancer [92], and renal cell carcinoma [93]. Liao and Peng demonstrated that the expression level of miR-206 increases in lung cancer cells and NSCLC tissues. It negatively regulates Coronin-1C (CORO1C) and then restrains the proliferation, migration, and invasion of A549 cells. CORO1C is a WD-repeat protein and is overexpressed in multiple types of clinically aggressive cancers, including glioblastoma and gastric cancer (Fig. **7**). The loss of CORO1C significantly represses cell invasion and metastasis [94].

MiR-206 may play a different role in different samples, such as lung tissue and blood of male F344 rats. The results from the studies of Wu and colleagues illustrated different opinions about the miR-206 expression level in rat serum [95]. Researchers detected the levels of serum miR-206 in lung cancer tissues of rats induced by 4-(methylnitros-amino)-1-(3-pyridyl)-1-butanone (NNK). NNK is an environmental pollutant that is the principal lung carcinogen [96]. Compared to the normal individual, the level of serum miR-206 was significantly up-regulated in the early stage and decreased during the late stages of NNK-induced lung carcinogenesis, while miR-206 exhibited low expression in the lung cancer tissues of rats. MiR-206 expression is an independent prognostic factor for patients with lung cancer.

#### 4.2.4 MiR-206 in Gastric Cancer

Gastric cancer is the second leading cause of cancer-related death worldwide and one of the most common cancers of the digestive system. The incidence of gastric cancer is very high in East Asian countries [97]. The 5-year overall survival rate of patients with gastric cancer in stage I has reached 90%, but less than 5% in stage IV. This implies that the prognosis in patients with gastric cancer has affiliation with the cancer stage [98]. Metastasis may be one of the deadliest threats to patients with gastric cancer [99]. In developing countries, including China, despite more advanced progress in chemotherapy and surgical techniques, the 5-year overall survival rate of patients with gastric cancer is still lower than 40% [100].

In agreement with previous studies, miR-206 plays important roles in tumorigenesis and tumor progression of various human malignancies. Research indicates that the level of miR-206 was noticeably decreased in advanced patients with gastric cancer compared to normal people [101]. In postoperative follow-up patients with gastric cancer, the five-year survival rate of gastric cancer patients with a high level of miR-206 has reached 80.6%, whereas the rate for patients with a low miR-206 expression is 57.7%. The results are sufficient to justify the importance of miR-206, indicating that it might be a potent prognostic marker for patients with gastric cancer in the near future (Fig. **7**).

#### 4.2.5 MiR-206 in Renal Cell Carcinoma

Renal cell cancer (RCC) is a heterogeneous disease (an epithelial tumor) derived from the proximal tubules of nephrons and is one of the most lethal types of cancer in adults [102]. The largest risk factor for RCC is genetic risk and histopathology [103]. The incidence of RCC in malignance has increased to 4% in the past five years [104]. Despite more advanced treatments, such as surgical resection for RCC, the rate of postoperative recurrence is relatively high. Approximately 30% of RCC patients have a recurrence and metastasis within 3 years [105].

Guo *et al.* found that miR-206 was downregulated in RCC tissues of patients undergoing radical nephrectomy as compared to non-cancerous tissues, and exogenous miR-206 (miR-206 mimics) remarkably inhibited RCC cell migration and invasion [93]. Research groups of Chao Wei made a similar point that miR-206 inhibited RCC cell growth partly by targeting G-associated kinase (GAK), a master regulator of tumor proliferation and metastasis [106]. Hence, miR-206 may be a hopeful sign as a potential therapeutic target for RCC (Fig. **7**).

### 4.3 MiR-206 in Tumors of Other Organs Besides Vital Organs of the Human Body

Colorectal cancer (CRC) is one of the most common malignant tumors, and the death rate is very high [107]. CRC accounts for the higher proportion in the numbers of all cancers and deaths. The most important cause of CRC patients’ death is due to metastasis [108]. Recently, Park *et al.* reported that miR-206 was down-regulated in human CRC tissues and cell lines from the Biobank of Chonbuk National University Hospital. Furthermore, miR-206 inhibited transmembrane 4 L six family member 1 (TM4SF1) expression *via* the binding of the TM4SF1 3′-UTR and suppressed cell proliferation, migration, and invasion in prostaglandin E2 (PGE2)-induced cells. The research provided further evidence that miR-206 had a protective effect on PGE2-induced colon carcinogenesis [109].

Furthermore, miR-206 may serve as a tumor suppressor in bladder cancer. Bladder cancer (BC) is the most common malignant tumor in the urinary system, and it ranks fourth among malignant tumors in males, especially in European and American countries [110]. The occurrence and development of bladder cancer are chronic and complicated processes. It is caused by multiple factors and multi-steps, such as smoking, prolonged exposure to radiation from electronic products, and hereditary factors [111]. Cao and their colleagues indicated that miR-206 had a negative correlation with the RNA component of mitochondrial RNA-processing endoribonuclease (RMRP) in BC tissues of patients. The overexpression of RMRP was found to cause bladder cancer metastasis as compared to adjacent tissues [112]. The higher expression of RMRP could promote the proliferation, migration, and invasion of BC cell lines *via* inhibiting the level of miR-206 as a sponge, exogenous miR-206 (miR-206 mimic) restrained the invasion of BC cell lines *via* binding with the RMRP 3′-UTR. MiR-206 may be a potential therapeutic target for BC (Table **2**).

### 4.4 Natural Products Regulate miR-206 in Disease

Ginkgo biloba extract (EGb) is one of the oldest living tree species in the world. Two major constituents of flavonoids and terpene lactones were extracted from the dried leaves of EGb. EGb was proven to be one of the most frequently investigated herbal medicines for enhancing cognition and alleviating neurodegenerative dementia [113]. The EGb administration alleviated the learning and memory deficits in behavioral experiments in the scopolamine (SCO)-induced AD model of rats and decreased the levels of miR-206-3p in the hippocampus of rats.

Cumin (*Cuminum cyminum* L.) is a member of the Apiaceae family; cumin is well known for its role as a spice used in Indian cuisine and for flavoring curries. Furthermore, the major phytochemical constituents of cumin are cuminaldehyde and cymene, which have been proven to stimulate the activities of cytochrome p450s, aryl hydroxylase, and N-demethylase in rats [114]. The cumin powder diet reversed the expression levels of miR-206 that were highly modulated by 17ß-estradiol (E2) treatment in August Copenhagen Irish (ACI) rats with breast cancer [115].

Astragalus polysaccharide (APS) is the main active ingredient derived from the traditional Chinese medicine astragalus, which has a variety of important biological activities. Astragalus polysaccharide regulated anticancer and immunomodulatory effects. Among them, APS plays an important role in immune regulation [116]. MiR-206 in bone tissues of patients with steroid-induced osteonecrosis of the femoral head (SONFH) was markedly elevated compared to normal bone tissue. APS promoted autophagy and inhibited apoptosis in the SONFH cell model by inhibiting the high expression of miR-206, while miR-206 mimics reversed the effect of APS [117]. It showed that APS could regulate apoptosis by regulating the expression of miR-206 in SONFH disease. Hence, miR-206 was found to be a useful drug target for SONFH patients (Table **3**).

## CONCLUSION AND FUTURE PERSPECTIVE

Non-malignant diseases, such as skeletal muscle maldevelopment, Alzheimer's disease, and depression, threaten normal human physiological and psychological health. Over the years, although many drugs have been developed to target specific parts of the disease pathways, they result in poor treatment, recurrence of the disease, and severe complications. The incidence of cancer is very high and has surpassed all other diseases, and up to now, it has become the leading cause of death worldwide. The new trends suggest that the main cause of cancer mortality could be attributable to the aging population, as the incidence of most cancers increases dramatically after 65 years of age [118]. The People's Republic of China has stepped up to the rank of aging countries. According to statistics, the proportion of older adults over 60 years old increased from 13.3% in 2010 to 18.7% in 2020 and might even be able to reach 35% by 2050. As may easily be imagined, the cancer burden would increase the burden on society accordingly [119]. Although scientists have made a major breakthrough in the treatment of cancer, bringing great hopes to those patients, the therapeutic effect is still far from satisfactory. It is urgent to find effective ways of preventing and treating cancer.

MicroRNAs are a class of small non-coding, single-stranded RNAs (ribonucleic acids) with a composition of approximately 20-24 nucleotides in length, which play important roles in many vital physiological and pathological processes through their potential to regulate the expression of any RNA. The biogenesis of miRNAs is a complicated system engineering. The first advance is the synthesis of a relatively long structured primary transcript (pri-miRNA) in the nucleus, then pri-miRNA is processed into an intermediate length hairpin precursor miRNA (pre-miRNA) with the help of the proteins Drosha and DGCR8 and then exported into the cytoplasm, at last Dicer cleaves the pre-miRNA to produce the mature miRNA in the cytoplasm.

This review article highlights the importance of miR-206 in the development of non-cancerous diseases (skeletal muscle injuries, AD, and depression) and cancer (hepatocellular carcinoma, lung cancer, colorectal carcinoma, *etc*.). In the current review, we summarized the role of miR-206 in both malignant and non-malignant situations and explained its possible implications for treatment in clinical practice. The roles of miR-206 in cancer can be categorized into two types: tumor-suppressor and tumor promoter. Investigating the role of miR-206 in cancer therapy also reveals that complicated signaling networks (miR-206 affects certain targets or different molecular pathways) are involved in this case, and miR-206 seems to inhibit tumor spread and metastasis in some cancers.

In addition, this review also explored the effects of natural medicine on miR-206 in some kinds of diseases. Natural medicine has a good prospect of development with the preponderance of low toxicity, low side effects, and high safety. Therefore, some natural medicine would be a nice choice to regulate the abnormal expression of miR-206 in the disease. For example, miR-206 is an effective target for Astragalus polysaccharide treatment in SONFH patients; APS could regulate apoptosis by regulating the expression of miR-206 in SONFH disease [117]. It is relatively rare to find some studies about the effects of natural medicine on miR-206; therefore, more research should be conducted in the future.

This review also has some limitations; for example, main attention was given only to the relationship between miR-206 and diseases. It is still unclear whether there are any other miRNAs involved in human disease and, if so, whether they also work through complicated signaling networks. Furthermore, specific miRNAs may not be used as therapeutic targets or produce effective therapeutic effects because epigenetic processes, including DNA methylation and histone modification, may be involved in the pathological mechanisms of disease.

Moreover, the mode of miRNA administration is discrepant on different diseases; it also depends on forms of modification on cellular miRNA expressions, such as synthetic miRNAs (miRNA mimics, miRNA agomir), oligonucleotide-based miRNA inhibitors (AntagomiR), and recombinant expression vectors carrying miRNA encoding sequences (adeno-associated virus) [120]. For example, Guan and his research group has confirmed that genetic overexpression of miR-206-3p (brain stereotactic injection of AAV-miR-206-3p or nasal inhalation of AgomiR-206-3p) aggravated the depression-like behaviors and neurogenesis damage of normal mice, while genetic knockdown of miR-206-3p (brain stereotactic injection of AAV-siR-206-3p or nasal inhalation of AntagomiR-206-3p) relieved the neurological damage and produced antidepressant effects in behavioral tests in depression model of mice [28]. Besides systemic applications *via* injection and infusion, advanced strategies emerge for miRNA-based drug administration *via* inhalation schemes (nasal delivery) [121], implantable 3D matrices, and intake *via* food [122].

The dosage of miRNA administration in disease is a problem that should be paid attention to in the medical domain. Due to any chemically modified RNA (exogenous RNA), the dosing of miRNA therapeutics can usually not be more than the physiological range of endogenous miRNA expression. Otherwise, it can consequently cause unpredictable off-target effects [123, 124]. N Narayan and L Morenos research group indicated that manipulated miRNA (miR-155) expression level could cause outcomes that are contrary to the therapeutic objectives in murine acute myeloid leukaemia (AML) models, which depends on the dosage of the highest miR-155 expression levels [125]. Hence, the dosing of miRNA therapeutics has to be kept within reasonable limits to induce a therapeutic effect, which may have important therapeutic implications. Further theoretical and empirical research works and experimental studies are needed in these fields.

However, recent advances have been made, such as miRNA interactions in disease. For example, in non-cancerous liver cells, miR-21 increases the expression of miR-21-targeted gene programmed cell death 4 (PDCD4), thus controlling cell proliferation. Conversely, if miR-122 regulation is deregulated, miR-21 expression increases, leading to a decrease in PDCD4 levels and thus contributing to a cancer phenotype [126, 127]. Moreover, miRNA interactions could be profound, and the community must be mindful of the effects of miRNA networks in studies pertaining to the role of miRNAs in cancer and beyond and their application in therapeutics.

To sum up, the miRNA therapeutic method is very promising to be applied to clinical practice; while we are only at the beginning of a research process, miRNA may be a new diagnostic biomarker for the disease. These findings increase our understanding of the pathogenesis of the disease and guide future treatment.

## Figures and Tables

**Table 1:** Aberrant miRNAs reported in human disease.

**miRNA**	**Change**	**Tissue or Serum**	**Regulatory Role**	**Disease**	**Targets**	**References**
miR-206	Up-regulation	Serum	Promote myoblast differentiation and regeneration	Skeletal muscle development	mTOR, TGF-β	[41, 46]
miR-206	Up-regulation	Serum, temporal cortex, CSF	Promote AD	AD	BDNF	[49, 54]
miR-206-3p	Down-regulation	Brain tissues	Promote AD	AD	BDNF	[60]
miR-206	Up-regulation	Hippocampus	Promote depression	Depression	BDNF	[66]
miR-206	Down-regulation	Hepatic tissue	Inhibit hepatocellular carcinoma cell growth	Hepatocellular carcinoma	G6PD	[75, 76]
miR-206	Up-regulation	Plasma	Promote hepatic carcinoma	Hepatic carcinoma	-	[77]
miR-206	Up-regulation	Heart tissue	Alleviating the heart failure	Heart disease	TIMP-3	[82]
miR-206	Down-regulation	Heart tissue	Increased cardiomyocytes apoptosis	Heart disease	PTP1B	[85]
miR-206	Up-regulation	lung cancer tissue	Inhibition of lung cancer cells	Lung cancer	CORO1C	[94]
miR-206	Down-regulation	serum, lung cancer tissues	Promote lung cancer	Lung cancer	NKK	[95]
miR-206	Down-regulation	adjacent mucosa	Promote gastric cancer	Gastric cancer	-	[101]
miR-206	Up-regulation	RCC specimens	Inhibition of RCC	Renal cell cancer	GAK	[106]

**Table 2:** Role of miR-206 in tumors of other organs besides internal organs.

**miRNA**	**Change**	**Tissue or Serum**	**Regulatory Role**	**Disease**	**Targets**	**References**
miR-206	Up-regulation	CRC tissues and cell lines	A protective effect on carcinogenesis	Colorectal cancer	PGE2	[109]
miR-206	Up-regulation	BC tissues	Inhibition of bladder cancer	Bladder cancer	RMRP	[112]

**Table 3:** Natural products regulate miR-206 in disease.

**Natural Products**	**miRNA**	**Change**	**Regulatory Role**	**Disease**	**Targets**	**References**
*Ginkgo biloba* extract	miR-206-3p	Down-regulation	A protective effect on AD	AD	PSD95	[113]
Cumin	miR-206	Down-regulation	Inhibition of mammary tumorigenesis	Breast cancer	ERα	[115]
Astragalus polysaccharide	miR-206	Down-regulation	Inhibition of steroid-induced osteonecrosis of the femoral head	SONFH	HIF-1α/BNIP3	[117]

## LIST OF ABBREVIATIONS

ACI: August Copenhagen Irish
AD: Alzheimer’s Disease
AMI: Acute Myocardial Infarction
AML: Acute Myeloid Leukaemia
APS: Astragalus Polysaccharide
BC: Bladder Cancer
BDNF: Brain-derived Neurotrophic Factor
CORO1C: Coronin-1C
CRC: Colorectal Cancer
CSF: Cerebrospinal Fluid
DGCR8: DiGeorge Syndrome Critical Region Gene 8
EGb: *Ginkgo biloba* Extract
EVs: Extracellular Vehicles
G6PD: Glucose-6-phosphate Dehydrogenase
GAK: G-associated Kinase
GATA4: GATA-binding Protein 4
GH: Group Housing
HCC: Hepatocellular Carcinoma
HDAC4: Histone Deacetylase 4
HF: Heart Failure
HMGB1: High Mobility Group Box-1 Protein
I/R: Ischemia/Reperfusion
LV: Left Ventricular
MCI: Mild Cognition Impairment
MiR-206: MicroRNA-206
mPFC: Medial Prefrontal Cortex
mRNA: Messenger RNA
mTOR: Mammalian Target of Rapamycin
myomiR: Muscle-specific miRNA
NNK: 4-(methylnitrosamino)-1-(3-pyridyl)-1-butanone
NSCLC: Non-small Lung Cancer
PDCD4: Programmed Cell Death 4
PGE2: Prostaglandin E2
piRNA: PIWI-interacting RNA
Pol II: Polymerase II
pri-miRNAs: Primary miRNA Transcripts
PS: Pregnant Stressed
qPCR: Quantitative Polymerase Chain Reaction
RanGTP: Ran Guanosine Triphosphate
RCC: Renal Cell Cancer
RISC: RNA-induced Silencing Complex
RMRP: RNA-processing Endoribonuclease
SCO: Scopolamine
SI: Social Isolation
siRNA: Small Interfering RNA
SONFH: Steroid-induced Osteonecrosis of the Femoral Head
TGF-β: Transforming Growth Factor Beta
TIMP-3: Tissue Inhibitor of Metalloproteinase 3
TM4SF1: Transmembrane 4 L Six Family Member 1
TMPD: 2,3,5,6-tetramethyl-p-phenylenediamine
tsRNA: Transfer RNA-derived Small RNA
UTRs: Untranslated Regions
WHO: World Health Organization
YAP: Yes-associated Protein
